# Whether and how tourism industry upgrading promotes employee social upgrading? Insights from China

**DOI:** 10.1371/journal.pone.0331022

**Published:** 2025-09-03

**Authors:** Tao Lin

**Affiliations:** School of Business Administration, Chongqing Technology and Business University, Chongqing, China; Indian Institute of Management Bodh Gaya, INDIA

## Abstract

In response to the decent work agenda in the United Nations Sustainable Development Goal 2030, an increasing body of studies has focused on social upgrading in developing countries. Numerous studies have explored whether and how economic upgrading promotes social upgrading in various industries and regions. However, few studies have examined the employee social upgrading resulting from the upgrading in the tourism industry. Moreover, most existing studies have adopted a qualitative approach, with limited quantitative research exploring the economic-social upgrading relationships. Using data from a survey conducted in China in 2024, this study examines whether and how tourism industry upgrading promotes social upgrading among related employees, as well as the role of governance environment in this relationship, employing a structural equation modeling (SEM) approach. The modeling results highlight the significance of tourism industry upgrading in promoting social upgrading of tourism industry employees. Additionally, the local governance environment is found to significantly moderate the relationship between tourism industry upgrading and employee social upgrading. This study contributes to the literature on economic and social upgrading by providing empirical evidence from the tourism sector and emphasizing the importance of governance in facilitating social improvements.

## 1. Introduction

For a long time, under the assumption that economic upgrading naturally leads to social upgrading, most studies in developing countries have focused on how to promote economic upgrading to generate greater value-added, rather than examining its social consequences [1]. Responding to the decent work agenda outlined in the UN Sustainable Development Goal 2030 (SDG 2030), which focuses on promoting sustained and inclusive economic growth, the concept of social upgrading was introduced in 2011 [2,3], which has spurred the emergence of studies on social upgrading. The automatic transformation theory in neoclassical economics posits that economic upgrading—such as industrial innovation and value-added growth—automatically enhances worker welfare through the “employment compensation effect,” including high-skilled job creation, wage increases, and improved social security [4]. Marxists hold that the profit-driven nature of capital makes enterprises tend to cut labor costs, which limits the social upgrading of local employees [5]. Numerous studies have empirically explored the effects of economic upgrading on social upgrading across various industries in different regions, including garment, electronics, timber, logistics, and assembly [6–8], and supported the automatic transformation theory. They found that different types of upgrading, such as process upgrading and product upgrading, can enable firms to produce more efficiently and explore new product lines, thereby promoting social upgrading [9,10]. However, some other studies have supported the Marxist perspective to a certain extent. They reported that the improvement of efficiency brought by industry upgrading can sometimes lead to intensified competition in job market and result in social downgrading of employees, such as the absence of work contracts, lower wages, and longer working hours [1,11,12].

Furthermore, findings from previous studies suggest that the impacts and underlying mechanisms through which economic upgrading affects social upgrading exhibit significant heterogeneity across regional, industrial, and demographic characteristics [6,11,13–16]. Additionally, researchers have identified varying governance environments as key determinants of diverse social upgrading outcomes across regions and industries [12,17,18]. Though numerous studies have empirically explored the economic-social upgrading relationships in various manufacturing industries, limited attention has been devoted to the social upgrading outcomes of upgrading in the tourism industry. Given significant differences between tourism and manufacturing industries in terms of employment structure, working contents, business models, governance structures, and other aspects, findings on economic-social upgrading relationships from manufacturing industries in other countries cannot be directly generalized to the tourism industry in China. Moreover, most previous studies have used a qualitative approach to examine these economic-social upgrading relationships, while few have quantitatively examined these relationships or the role of governance environments. Thus, based on survey data from the tourism industry in Chongqing, China, this study aims to quantitatively investigate whether and how tourism upgrading drives social upgrading among employees, as well as how the local governance environment influences social upgrading outcomes.

The tourism industry is a labour-intensive and comprehensive sector characterized by high employment capacity. Many jobs in the tourism industry have low requirements for educational qualifications and skills; consequently, tourism has become an important sector for employing low-skilled populations in developing societies. However, due to tourism’s high seasonality, temporary employment is widespread in the tourism industry, and employment stability within the sector remains relatively low. This results in widespread inadequate access to social security among employees in the tourism industry. Furthermore, compared to other manufacturing sectors, the tourism industry generates less tax revenue for local governments, leading to generally insufficient attention from local authorities toward the sector’s development. However, during the post-pandemic period, the weakening of consumer demand (driven by economic stagnation) and the intelligent development of manufacturing industries have led to a contraction in manufacturing employment. Promoting the recovery and development of the tourism industry has become a critical strategy for many governments to boost employment and advance social development. Therefore, it is urgent to explore the relationship between tourism upgrading and social upgrading to facilitate social upgrading in developing countries.

Chongqing, a provincial-level administrative region in southwestern China, provides a uniquely significant context for investigating tourism upgrading and social upgrading. With a population exceeding 32 million, Chongqing is classified as a moderately developed region in terms of national economic and social development. Boasting a diverse geographical environment and abundant tourism resources, Chongqing’s tourism industry ranks among the top nationally. In recent years, promoting tourism development has been regarded as a key policy for the Chongqing municipal government to promote rural revitalization and social development. Furthermore, as a centrally administered municipality in western China, Chongqing serves as a key pilot region for implementing regional policies and possesses greater policy flexibility. Therefore, Chongqing serves as an ideal context for exploring how tourism upgrading drives social development and the role of the local governance environment in this relationship. This study will provide novel empirical evidence to advance theoretical debates on the interplay between economic and social upgrading, as well as their underlying mechanisms.

The rest of the paper is organized as follows: The next section reviews the literature on economic upgrading and social upgrading, as well as tourism upgrading and its social consequences, and illustrates the conceptual framework of this research. Section 3 provides details on the data and variables used in the study. Section 4 presents the model design and the modeling results. The final section draws conclusions, and discusses the implications of the findings.

## 2. Literature review and conceptual framework

### 2.1 Economic upgrading and social upgrading

Economic upgrading, also known as industrial upgrading, is typically defined as the capacity and process of firms and industries to create higher-quality products and improve competitiveness by enhancing their production processes, efficiency, or products, or transitioning into more skill-intensive activities [2,3]. It is commonly distinguished into four types: product upgrading (developing higher-value products), process upgrading (improving production efficiency), functional upgrading (advancing in the value chain), and chain upgrading (shifting to higher-value industries) [17]. Social upgrading, on the other hand, refers to the process of enhancing the basic rights of workers as social participants and improving their employment quality [2]. It is a key component of the decent work agenda advocated by the United Nations and the International Labour Organization. In recent years, with the growing emphasis on the International Labour Organization’s Decent Work Agenda, an increasing number of scholars have explored whether and how economic upgrading leads to social upgrading.

Findings from existing studies generally suggest a complex relationship between economic and social upgrading. Although social upgrading often co-occurs with economic upgrading, economic upgrading does not necessarily lead to social upgrading; it can sometimes result in social downgrading [11,12]. Moreover, the impacts of economic upgrading on social upgrading vary significantly across different countries, industries, and labor groups [1,19,20]. Using statistical data, Bernhardt & Pollak (2016) conducted a comparative analysis of the relationship between economic upgrading and social upgrading in four manufacturing industries across 35 developed and developing countries [6]. Their findings revealed an overall positive correlation between economic and social upgrading, but also identified instances where industrial upgrading was associated with social downgrading. Lund-Thomsen et al. (2012) analyzed the differences in economic-social upgrading relationships among China, Pakistan, and India and found that Chinese factories were the first to adopt mechanized production, leading to industrial upgrading and resulting in improved wages and social security for workers, outpacing the other two countries [21]. However, issues such as longer working hours and a lack of social dialogue were also observed alongside these industrial upgrades for Chinese workers. Other studies have explored the economic-social upgrading relationships by focusing on specific industries and worker groups. For example, Rossi (2013) focused on the Moroccan apparel industry and revealed that process upgrading contributed to improvements in fundamental social rights, such as reduced overtime and enhanced working conditions [11]. Product upgrading provided fixed workers with opportunities for skill training, and functional upgrading led to enhancements in both fundamental and protective social rights for fixed workers. However, temporary workers in packaging and warehousing roles faced social downgrading, such as lower wages, excessive overtime, and a lack of protective social rights. Based on a case study of producers and workers in the African horticulture sector, Barrientos et al. (2016) found that participation in global value chains compels horticulture companies to upgrade their production processes due to competitive pressures, which promotes social upgrading for some workers [14]. However, temporary workers generally face social downgrading characterized by low wages, lack of labor contracts, job insecurity, and insufficient social protection. Similar results are also observed in the business process outsourcing (BPO) industry in South Africa, where economic upgrading among BPO firms was found to lead to both social upgrading and downgrading among contact center workers [19]. Wang et al. (2020) also reported social downgrading caused by economic upgrading for rural migrant workers in the Pearl River Delta region of China [1]. They argue that economic upgrading tends to cause a pushing-out effect, such as increasing unemployment or working intensity, and increasing living costs for rural migrant workers.

### 2.2 Governance environment and social upgrading

A number of studies have paid attention to the factors underlying the uneven social outcomes of economic upgrading among different countries, industries, and labor groups, and argue that the governance environment plays a crucial role in shaping these uneven social outcomes [17,22]. The governance environment refers to the rules, norms, and processes that regulate economic activities and distribute resources. Existing research identified three types of governance: social governance, private governance, and public governance [18]. Social governance is mainly led by civil society organizations, such as non-governmental organizations, trade unions, and multi-stakeholder organizations. It typically lacks enforceability, and its effect on social upgrading is often constrained by market conditions and institutions at the national and local levels [23]. Private governance refers to how companies regulate suppliers in their supply chains through codes of conduct, corporate social responsibility initiatives, and oversight, thereby promoting improvements in employment quality among suppliers at various levels, leading to social upgrading [24]. Public governance is dominated by public actors, including local governments at various levels and supranational organizations. This form of governance is characterized by enforceability, manifested in formal government regulations, laws, and bilateral or multilateral trade agreements, which significantly impact social upgrading [20].

The governance environment can influence social upgrading in several ways. For example, effective labor regulations and enforcement can better protect workers’ rights and ensure that upgrading leads to improvements in working conditions and wages [2]. Corporations with strong social responsibility consider a wide range of social, environmental, and human rights issues in their core business practices, focusing on ensuring fair wages, safe working conditions, and providing opportunities for skill development [17]. By doing so, companies contribute to enhancing the social upgrading of their workforce. On the other hand, weak governance may also lead to social downgrading. For instance, lax labor laws and enforcement can allow employers to exploit workers in the name of competitiveness [12].

### 2.3 Tourism industry upgrading and social upgrading

Tourism is a significant sector for many economies, particularly in developing countries, where it serves as a key source of employment and a major driver of economic growth [25]. For a long time, based on the neoclassical economics thesis of automatic transformation—which posits that tourism development naturally drives social progress—numerous studies on tourism upgrading have primarily focused on promoting sustainable tourism development and enhancing tourism’s value-added [26–29]. However, little attention has been paid to its social consequences, particularly in terms of social upgrading for tourism employees. The few exceptions include Christian (2016), who has examined the impact of global production networks in tourism on local social upgrading in Kenya and Uganda [30]. Findings suggest that governance relationships between tour operators and accommodation firms directly affected social upgrading outcomes for hotel workers and indirectly influenced those for excursion workers. From a political economy perspective, Bianchi & de Man (2021) argue that in the development of capitalist tourism, the commercialization of labor and exploitation of natural resources lead to systemic injustice in local areas [31]. They found that although tourism growth may create job opportunities, it is often accompanied by issues such as land dispossession, ecological damage, deteriorating working conditions, and rising inequality. In addition, although not directly addressing the social upgrading issue, numerous studies have examined the diverse impacts of tourism development on local communities, such as poverty reduction [32,33], social injustice [34], income inequality [35], and decent work [36], among others.

Despite significant progress in understanding the social consequences of tourism development, many issues remain to be explored in future research. An important question that remains underexplored in the literature is whether and how tourism industry upgrading impacts the social upgrading of tourism employees, as well as the role of the governance environment in shaping these upgrading outcomes. Tourism is widely regarded as a key driver of economic and social revitalization in developing countries [25]. Therefore, this study examines the nuanced relationships between tourism industry upgrading and social upgrading, as well as the underlying mechanisms, aiming to generate deeper insights and harness the potential of tourism upgrading to drive social upgrading.

### 2.4 Conceptual framework

**Fig 1:**
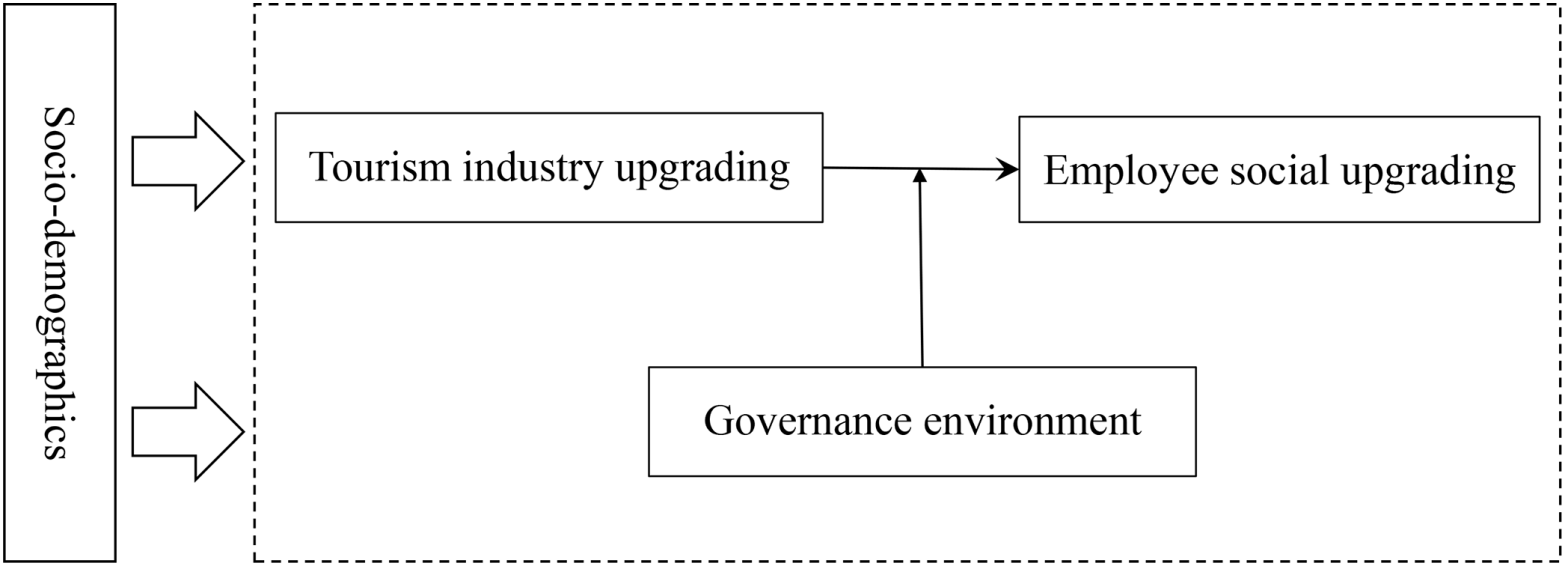
Conceptual framework.

Fig 1 illustrates the conceptual model of this study. As previously reviewed, economic upgrading significantly impacts social upgrading [6,20]. Therefore, following the automatic transformation theory and previous studies, it is reasonable to assume that tourism industry upgrading may significantly and positively influence social upgrading among related workers. Specifically, tourism product upgrading, facility upgrading, service upgrading, and structural optimization in scenic areas may enhance tourist accessibility, travel experiences, and the scenic area’s competitiveness, thereby increasing tourist numbers and business performance [37,38]. Increases in tourist numbers and consumption may drive prosperity in the tourism employment sector [39], such as more job opportunities, higher wages, and improved benefits (e.g., social insurance) and stronger labor rights. As key stakeholders, employees may also participate in more social dialogues during this process. Therefore, it is hypothesized that tourism facility upgrading, service upgrading, and structural optimization may significantly and positively influence the four dimensions of social upgrading: employment improvements, social insurance, labor rights, and social dialogue.

Moreover, findings from several studies suggest that the uneven outcomes of economic upgrading on social upgrading may be largely shaped by the governance environment [8,17]. It is thus reasonable to hypothesize that the influences of tourism industry upgrading on social upgrading may depend on the local governance environment. In other words, the local governance environment significantly moderates the effects of the four dimensions of tourism industry upgrading on social upgrading. Furthermore, personal socio-demographics are included as exogenous variables, as these factors may shape related workers’ job choices and social upgrading outcomes.

## 3. Data and variables

### 3.1 Data

The data used in this study were obtained from a sample survey conducted from June to September 2024 in Chongqing, China. The study is approved by the review committee of the School of Business Administration, Chongqing Technology and Business University (approval number 23SKGH176−1). Before the survey, investigators communicate with the respondents, explaining the purpose, process, and confidentiality measures of the study. Once respondents agreed to participate in the survey and signed the written informed consent, investigators provided guidance or assistance to help them complete the questionnaires. All data collected were strictly confidential, and during the research process, all data were analyzed anonymously to prevent any possible disclosure of personal identity.

Respondents were recruited from 16 scenic areas across 16 districts or counties (out of a total of 38 districts or counties in Chongqing). The sampling of scenic areas was conducted using a stratified sampling approach, considering the types of scenic areas and their geographical locations, given that different types of scenic areas and different locations may significantly influence the tourism development of these scenic areas. Specifically, scenic areas in Chongqing were classified into four categories: urban scenic areas, natural scenic areas, rural agricultural scenic areas, and rural ethnic culture villages. Besides, according to geographical location, Chongqing is composed of four regions: central urban area, the metropolitan area outside the central urban area, northeastern region and southeastern region, and the central urban area includes 9 districts, the metropolitan area outside the central urban area includes 13 districts, and the northeastern region and southeastern region of Chongqing consist of 10 districts and 6 districts respectively. To make sure the representativeness of our sample, the scenic areas and districts were carefully sampled to include all the most typical and famous scenic areas within the four regions of Chongqing. Accordingly, a total of 16 scenic areas from 16 districts or counties were selected, with three in the central urban area, six in the metropolitan area and seven in the northeastern and southeastern regions. Finally, investigators approached and recruited potential respondents in each scenic area through face-to-face interviews at the workplaces in the scenic areas. On average, each respondent took 5–10 minutes to complete the questionnaire. Prior to the formal survey, a pilot survey was conducted with 30 questionnaires, and minor revisions were made to the questionnaire based on the feedback. In total, 462 questionnaires were collected, but 11 of them were invalid due to missing values or repetitive responses to most questions. These invalid questionnaires were excluded, resulting in a final sample of 451 cases for this study.

The data collected and used in this study encompass information on the tourism industry upgrading of scenic areas, social upgrading of respondents, local governance environment, and socio-demographic characteristics. Tourism industry upgrading data were collected by asking respondents to rate their scenic area on a 5-point Likert scale, ranging from “strongly disagree” to “strongly agree,” in response to statements regarding tourism industry upgrading. On average, the respondents had worked in their districts for 6.2 years and in their current job for 4.2 years, with 90% having worked for more than 2 years in their current job. Therefore, most of them have witnessed the developments in the tourism industry before the survey and have adequate understanding of the upgrading situation of the local tourism industry. Social upgrading data were also collected through subjective measures. Social upgrading typically includes four dimensions: (1) Employment, which involves providing workers with full employment opportunities, adequate remuneration, and a safe and healthy working environment; (2) Social insurance; (3) Labor rights, including protections against discrimination, forced labor, and child labor; and (4) Social dialogue, which refers to the formal expression of workers’ rights, their participation in trade unions, and involvement in negotiations regarding work-related matters [19]. Based on these dimensions, statements were designed, and respondents were asked to rate them using a 5-point Likert scale ranging from “strongly disagree” to “strongly agree.” The governance environment in industrial clusters includes public governance (government regulations), private governance (cooperatives responsibilities) and social governance (e.g., labor unions, NGOs) [17]. Given that there are few NGOs in China and labor unions at the government level are semi-governmental organizations while those at the firm level are led by enterprises [40], the local governance environment was measured solely through statements on the roles of local governments and tourism enterprises in regulating tourism development and labor rights—characterizing public governance and private governance, respectively. The contents of social governance, such as the roles of NGOs and labor unions, were not included.

### 3.2 Variables

Table 1 provides the definitions and descriptive statistics of the explanatory variables used in the models examining the effects of tourism industry upgrading on social upgrading and the role of the local governance environment. Following previous studies [41,42], tourism industry upgrading is characterized by four latent variables: product upgrading, facility upgrading, service upgrading, and structural optimization. Each of these four latent constructs is measured with five indicators, as shown in Table 1. To ensure the reliability of these indicators, the coefficient of intrinsic consistency was calculated (with Cronbach’s α equal to 0.867), indicating that our measures are reliable. Additionally, the results of the exploratory factor analysis (EFA) also suggested good validity of our indicators (with KMO equal to 0.849 and the p-value of Bartlett’s sphericity test less than 0.000). Following Anwar & Graham (2019), four variables were used to measure the four dimensions of social upgrading: employment improvement, social insurance, labor rights, and social dialogue [19]. With reference to previous studies [19,43], seven indicators are designed to measure these four dimensions, as shown in Table 1. The results of the reliability and validity tests of these indicators suggest that our measures are acceptably reliable (Cronbach’s α equal to 0.714) and valid (KMO equal to 0.766 and the p-value of Bartlett’s sphericity test less than 0.000). Following Gereffi & Lee (2016) and Golini et al. (2018), two latent constructs, Government Responsibility (GR) and Corporate Social Responsibility (CSR), characterizing public governance and private governance respectively, are used to measure the governance environment [8,16]. With reference to previous studies [8,17,25,44], four indicators were designed to measure the construct of Government Responsibility. The concept of CSR was widely understood from a stakeholder perspective and defined as the relationship of a firm with its stakeholders [45,46]. Scholars have identified four sets of stakeholders: employees, customers, government, social and non-social [45]. Since this study focuses on the social upgrading of tourism industry employees, the construct of CSR here was thus measured only from employee’s perspective. Since CSR involves the ethical and responsible treatment of its stakeholders by the enterprise [47], two indicators characterizing these two aspects of the tourism employees’ working enterprises were thus designed to measure the construct of CSR. Similarly, the test results of reliability and validity show that our measurement is feasible (Cronbach’s α equal to 0.754, KMO equal to 0.783, and the p-value of Bartlett’s sphericity test less than 0.000). Finally, six socio-demographic variables are included as control variables: age, gender, education, monthly income, local people, and working years. The scales of the latent variables are computed by averaging their indicators [48].

**Table 1:** Explanatory variables.

Category	Variable name	Indicators	Mean/%	Std.
Tourism industry upgrading (TIU)	Product upgrading (PU)	The development of new tourism products has expanded choices for travellers in recent years.	3.93	0.89
		In recent years, the quality of tourism products at attractions has been continuously improving.	3.81	0.87
		The number of high-quality hotels and restaurants near attractions has increased in recent years.	3.67	0.95
		Attractions are introducing tourism products that align with market trends based on visitor needs.	3.67	0.91
		Attractions are continuously developing unique tourism products based on local characteristics.	3.66	0.94
	Facility upgrading (FU)	In recent years, tourism transportation at attractions has become more convenient.	4.00	0.91
		The internet coverage at attractions has increased, providing more convenient access to the web.	3.80	0.91
		The number of public restrooms at attractions has increased, and they are kept cleaner and more sanitary.	3.72	0.97
		I acknowledge the investment and effectiveness in upgrading tourism facilities.	3.84	0.89
		In recent years, the renovation and upgrading of tourism facilities have met the needs of tourists.	3.76	0.93
	Service upgrading (SU)	In recent years, the quality of tourist services at attractions has been continuously improving.	3.86	0.84
		The attraction has intensified staff training, leading to an enhanced service awareness.	3.74	0.87
		In recent years, the occurrence of soliciting and overcharging tourists at the attraction has significantly decreased.	3.80	0.90
		The attraction places emphasis on enhancing tourists’ travel experiences and providing personalized services.	3.77	0.92
		The attraction utilizes advanced technology to enhance service levels, such as smart guides and online reservations.	3.91	0.89
	Structural optimization (SO)	In recent years, the attraction has seen a broader range of visitor sources, with an increase in the number of international tourists and a higher proportion of visitors from major cities.	3.77	0.88
		The ‘eating, accommodation, transportation, sightseeing, shopping, and entertainment’ services in tourist attractions fulfill tourist needs.	3.82	0.82
		The proportion of tourist spending on ‘sightseeing, shopping, and entertainment’ increases.	3.70	0.87
		The capability of integrated development between scenic tourism and cultural elements has strengthened.	3.61	0.91
		Scenic area tourism development emphasizes the harmony between resources and the environment.	3.63	0.89
Social upgrading (SU)	Employment improvement (EI)	Tourism-related job opportunities have increased in recent years.	3.56	0.98
		My salary/income has increased in recent years.	3.23	1.01
		The working environment and conditions have improved in recent years.	3.71	0.87
	Social insurance (SI)	I have medical and social insurance, and I am satisfied with them.	3.69	0.87
	Labor rights (LR)	I have not been forced to work, and the overtime hours and overtime pay are reasonable.	3.70	0.90
		I have not encountered discrimination or unfair treatment at work.	3.67	0.92
	Social dialogue (SD)	I am free to participate in union organizations, collective bargaining, and negotiations.	3.63	0.93
Governance environment (GE)	Government responsibility (GR)	Local governments provide significant policy support for the development of the tourism industry.	3.89	0.89
		Local governments allocate substantial financial investment to support tourism development.	3.90	0.87
		Local authorities place a strong emphasis on safeguarding the rights and interests of tourism workers.	3.73	0.84
		Local governments prioritize the interests and development of community residents in tourist destinations.	3.75	0.86
	Corporate social responsibility (CSR)	Tourism enterprises place importance on employees’ rights and welfare.	3.67	0.91
		Tourism enterprises possess a sense of social responsibility and service consciousness.	3.76	0.87
Social-demographics	Age	1(under 18 years old); 2(18–25 years old);3(26–35 years old); 4(36–45 years old); 5(46–55 years old); 6(above 55 years old)	3.32	1.22
	Gender	Male = 1	42.6%	–
		Female = 0	57.4%	–
	Education	1(junior high school and below); 2(high school and technical school); 3(junior college); 4 (undergraduate); 5 (postgraduate or above)	2.57	1.21
	Monthly income	Respondents’ after-tax monthly income (Yuan)：1(0–3000);2(3001–5000);3(5001–7000);4(7001–10000);5(10001–15000);6(15001–20000);7(20000 and above)	2.46	1.32
	Local people	Yes = 1	63.4%	–
		No = 0	36.6%	–
	Employment duration	Employment duration in current job (years)	4.18	4.44

## 4. Modeling results

### 4.1 Modeling approach

The conceptual model presented in Fig 1 is operationalized as a structural equation model (SEM), more specifically a path model. SEM is a modeling technique that simultaneously estimates complex relationships among a set of variables and has been widely used in social science studies [49,50]. Since our conceptual framework involves four dependent variables characterizing social upgrading, SEM is an appropriate and reasonable choice for this study. Moderating effects are frequently assessed by incorporating interaction terms between the moderator and the primary independent variable as extra predictors in the model [51]. In this research, a comparable modeling framework is employed to investigate the moderating role of the governance environment. Interaction terms frequently display high correlations with their corresponding main effects, which can result in multicollinearity challenges. To mitigate this issue, following Kuvaas (2008), the interaction terms were generated by centralizing the governance environment and tourism industry upgrading variables (adjusting their values by subtracting their means) prior to multiplication [48]. This centralizing process successfully lowers the Variance Inflation Factor (VIF) values to below 5 and raises the Tolerance values to above 0.2, effectively resolving the multicollinearity concern. Furthermore, centralizing the variables improves the interpretability of the findings.

AMOS (version 26) with the maximum likelihood (ML) estimator was used to estimate the model. A bootstrapping procedure (Bootstrap = 500) is also adopted when estimating the model to enhance the reliability of the estimates [50]. To evaluate the model fit, several commonly used fit indices were selected, including the chi-square value, the ratio of χ² to degrees of freedom, the Comparative Fit Index (CFI), the Normed Fit Index (NFI), the Root Mean Square Error of Approximation (RMSEA), the incremental fit index (IFI), and the goodness of fit index (GFI) [50,52]. Our model has a χ² value of 244.3with 94 degrees of freedom. The ratio of χ² to degrees of freedom is 2.60, CFI is 0.951, NFI is 0.928, RMSEA is 0.060, IFI is 0.955, and GFI is 0.956. Based on the cutoff values suggested (χ²/df < 5, CFI > 0.9, NFI > 0.9, RMSEA < 0.08, IFI > 0.9, GFI > 0.9) for a well-fitted model [50,53], these goodness-of-fit indicators suggest that our model fits the data reasonably well. In the following sections, the detailed modeling results will be discussed.

### 4.2 Effects of tourism industry upgrading on social upgrading

Table 2 presents the effects of tourism industry upgrading and the local governance environment on the social upgrading of tourism industry workers. Supporting our hypothesis, tourism industry upgrading is found to be a significant determinant of the social upgrading of tourism industry workers. Specifically, facility upgrading, service upgrading, and structural optimization are significantly and positively related to improvements in the employment of tourism industry workers. This indicates that facility upgrading, service upgrading, and structural optimization in tourism areas tend to lead to employment improvement for tourism industry workers. This is reasonable and easy to understand, as facility upgrading, service upgrading, and structural optimization tend to enhance the attractiveness of tourist spots, drawing more visitors and increasing consumption, which in turn may lead to an increase in jobs and salaries for related workers. Additionally, it is also found that facility upgrading and service upgrading tend to enhance social insurance coverage and satisfaction for tourism industry workers. Furthermore, facility upgrading, service upgrading, and the structural optimization of scenic areas are also found to have significant positive impacts on the labor rights of tourism industry workers, suggesting that tourism development tends to be conducive to the protection of the labor rights and interests of tourism industry workers. Finally, service upgrading is found to significantly enhance social dialogue. This is reasonable, as most service training in China is government-led and largely supported by companies and industry associations. These training sessions themselves serve as forums for local governments to dialogue with tourism-related enterprises and employees; therefore, service upgrading enhances social dialogue for tourism industry workers.

**Table 2:** Effects of tourism industry upgrading and governance environment on social upgrading.

From	To			
	Employment improvement (EI)	Social insurance (SI)	Labor rights (LR)	Social dialogue (SD)
Product upgrading (PU)	0.044	−0.005	−0.067	−0.062
Facility upgrading (FU)	**0.158** ^**a**^	**0.214** ^**a**^	**0.280** ^**a**^	0.061
Service upgrading (SU)	**0.159** ^**a**^	**0.233** ^**a**^	**0.207** ^**a**^	**0.400** ^**a**^
Structural optimization (SO)	**0.369** ^**a**^	−0.016	**0.212** ^**a**^	−0.021
Government responsibility (GR)	**0.100** ^**c**^	0.072	**0.140** ^**b**^	0.060
Corporate social responsibility (CSR)	0.046	**0.185** ^**a**^	**0.089** ^**c**^	**0.285** ^**a**^

Notes: ^a^significantly different from zero at p < 0.01; ^b^significantly different from zero at p < 0.05; ^c^significantly different from zero at p < 0.10 [55]. All effects are unstandardized.

However, no significant impacts of product upgrading are found on all four dimensions of social upgrading, indicating that product upgrading in scenic areas tends not to bring significant social upgrading for tourism industry workers. One possible reason behind these unexpected results may be the mismatch between the new demands generated by the product upgrading and the existing employment structure in the tourism industry. Specifically, the rapid development of smart tourism has made the product upgrading of scenic areas in Chongqing rely heavily on information technology (e.g., VR/AR interactive products, immersive experience products, smart scenic spots) in recent years. This kind of product upgrading has generated some demand for highly skilled employment (e.g., intelligent device operation, data analysis, creative design), while at the same time leading to a decline in the demand for traditional service positions (e.g., service staff, ticket seller, tour guide). For most existing low-skilled tourism industry employees, on the one hand, they are not competent for the high-skilled jobs that are generated by the product upgrading. On the other hand, due to the decline in low-skilled positions caused by product upgrading, they may face greater competition for employment after these product upgrades. Therefore, the product upgrading in tourism areas tends not to bring any significant social upgrading to the tourism industry employees, especially those with low educational attainment and low-skilled tourism employees. In addition, the findings further reveal that facility upgrading has no significant effect on social dialogue, whereas structural optimization shows no notable impact on either social insurance or social dialogue of tourism industry workers. This result is understandable. On the one hand, facility upgrading and structural optimization of tourism areas do not directly involve tourism workers’ social insurance, nor do they affect the communication process between workers, business owners, and the government. On the other hand, these initiatives are typically government- and capital-led, where authorities and businesses hold absolute decision-making power while employees lack institutionalized participation channels [54]. This power disparity substantially restricts the development of social dialogue among tourism workers. Overall, these findings may provide some evidence to support the Marxist viewpoint that tourism upgrading may not lead to social upgrading due to the profit-driven nature of capital. Nevertheless, these findings require verification through future studies.

### 4.3 Effects of governance environment on social upgrading

Referring to the effects of the governance environment, our findings support our hypothesis and align with those of previous studies (e.g., Gereffi & Lee, 2016; Lund-Thomsen et al., 2012) [17,21]. The governance environment was found to significantly and directly impact the social upgrading outcomes for tourism industry workers. Specifically, the responsibilities of the local government are positively related to improvements in employment and labor rights. This indicates that local governments that are more supportive of tourism development and more accountable for labor rights (demonstrating a stronger sense of government responsibility) tend to lead to an increase in tourism jobs, improvements in salaries, working conditions, and labor rights for tourism industry workers. Furthermore, corporate social responsibility significantly and positively impacts social insurance, labor rights, and social dialogue. This suggests that corporate social responsibility plays a significant role in the social upgrading of tourism industry workers. Employees working for companies with stronger corporate social responsibility are more likely to benefit from social upgrading as a result of tourism industry upgrading, particularly in areas such as social insurance, labor rights, and social dialogue.

Turning to the moderating effects of the governance environment, Table 3 presents the effects of interaction terms on social upgrading. As the table illustrates, five out of the eight interaction terms are statistically significant determinants of tourism industry workers’ social upgrading, providing strong evidence for our moderating hypothesis. Specifically, the interaction term “GR*FU” is significantly and positively related to employment improvement and social insurance. This suggests that the positive effects of facility upgrading on employment improvement and social insurance tend to be strengthened when the local government is more supportive of tourism development and more accountable for labor rights. In contrast, if the local government is less supportive of tourism development and less accountable for labor rights, the positive effects of facility upgrading on employment improvement and social insurance for tourism industry workers tend to be neutralized. Additionally, “GR*SU” is also found to have a significant positive effect on social insurance, indicating that a local government that is more supportive of tourism development and more accountable for labor rights tends to intensify the positive effect of structural optimization on social insurance. These results are easy to understand and “may” further demonstrate the important role of local government in shaping the social upgrading outcomes of tourism industry workers in China.

**Table 3:** Effects of interaction terms on social upgrading.

From	To			
	Employment improvement (EI)	Social insurance (SI)	Labor rights (LR)	Social dialogue (SD)
Government responsibility*Product upgrading (GR*PU)	−0.053	−0.130	−0.139	−0.108
Government responsibility * Facility upgrading (GR*FU)	**0.190** ^**b**^	**0.234** ^**c**^	−0.019	−0.101
Government responsibility * Service upgrading (GR*SU)	−0.111	**0.227** ^**c**^	−0.097	−0.030
Government responsibility * Structural optimization (GR*SO)	0.005	−0.182	0.108	0.013
Corporate social responsibility * Product upgrading (CSR*PU)	0.003	−0.113	−0.032	−0.012
Corporate social responsibility * Facility upgrading (CSR*FU)	**−0.154** ^**b**^	**−0.188** ^**c**^	0.119	0.103
Corporate social responsibility * Service upgrading (CSR*SU)	**0.226** ^**b**^	−0.019	0.136	0.129
Corporate social responsibility * Structural optimization (CSR*SO)	0.043	**0.563** ^**a**^	0.021	0.121

Notes: ^a^significantly different from zero at p < 0.01; ^b^significantly different from zero at p < 0.05; ^c^significantly different from zero at p < 0.10(Wang et al., 2011). All effects are unstandardized.

As for the moderating effects of corporate social responsibility (CSR), it was found that “CSR*FU” was negatively related to employment improvement and social insurance, suggesting that the positive effects of facility upgrading on employment improvement and social insurance tend to be neutralized for employees working in companies with stronger CSR. In contrast, the positive effects tend to be “more pronounced” for those working in companies with weaker CSR. This result “may” also indicate that small tourism business workers are more likely to benefit from tourism facility upgrading. This is understandable, as compared to large tourism companies, which usually have stronger CSR, small tourism businesses (typically with weaker CSR) are more flexible in responding to changes in the tourism market. Therefore, increases in jobs, salaries, and improvements in social insurance are more likely to occur in small businesses as a result of tourism facility upgrading. Moreover, “CSR*SU” is found to have a positive effect on employment improvement, suggesting that the positive effects of service upgrading on employment improvement tend to be intensified for those working in companies with stronger CSR. While for those working in companies with weaker CSR, the positive effects of service upgrading on employment improvement tend to be weakened. This is easy to understand since large tourism companies (typically with strong CSR) are more likely to provide systematic service trainings for their employees and promote their personal growth. Furthermore, “CSR*SO” is also found to be positively related to social insurance, suggesting that stronger CSR tends to intensify the positive effects of structural optimization on social insurance. This is reasonable because tourism enterprises with stronger CSR are usually more concerned about the welfare of their employees, while social insurance is usually an important component of employee welfare. Therefore, when the enterprises benefit from structural optimization, they are more inclined to share profits with employees, such as enhancing social insurance.

In addition, building upon prior studies (e.g., Loh et al., 2019; Sedera et al., 2017) [56,57], we seek to gain deeper insights into how the high, neutral, or low levels of the moderating variable (GR and CSR) influence the nature and magnitude of the effects exerted by tourism industry upgrading on social upgrading. The low, neutral, and high classifications were defined as follows: low was falling below the mean minus one standard deviation, neutral was at the mean, and high was above the mean plus one standard deviation. The results are presented in Table 4. As shown in Table 4, the moderators significantly altered both the nature and strength of the effects between tourism industry upgrading and social upgrading. Specifically, for varying levels of GR, the effects of facility upgrading on employment improvement and social insurance, as well as the effect of service upgrading on social insurance, varied. For workers with high levels of GR, the effect coefficients of facility upgrading on employment improvement and social insurance exceed 0.279 and 0.363, respectively, and the effect of service upgrading on social insurance exceeds 0.378. For those with low levels of GR, the corresponding effect coefficients are less than 0.037, 0.065, and 0.088, respectively—less than one-fifth of those in the high-GR group.

**Table 4:** The changes in the nature or strength of the relationships for different levels of governance environments.

Moderator	Relationships	Low-level (below the mean −1 SD)	Neutral level (the mean)	High-level (above the mean + 1 SD)
Government responsibility (GR)	Facility upgrading (FU)→ Employment improvement (EI)	<0.037	0.158	>0.279
	Facility upgrading (FU)→Social insurance (SI)	<0.065	0.214	>0.363
	Service upgrading (SU) →Social insurance (SI)	<0.088	0.233	>0.378
Corporate social responsibility (CSR)	Facility upgrading (FU)→ Employment improvement (EI)	>0.274	0.158	<0.042
	Facility upgrading (FU)→ Social insurance (SI)	>0.355	0.214	<0.073
	Service upgrading (SU)→ Employment improvement (EI)	<−0.011	0.159	>0.329
	Structural optimization (SO)→ Social insurance (SI)	<−0.054	0.369	>0.792

Note: Since mean value of the indicators of government responsibility and corporate social responsibility are adopt in our model, therefor the standard deviation for government responsibility is 0.637, and for corporate social responsibility is 0.751.

Turning to the moderating role of CSR, for corporate employees with low- and high-level CSR, the nature of the service upgrading-employment improvement relationship and the structural optimization-social insurance relationship differed. Specifically, the influence coefficient of the service upgrading-employment improvement relationship exceeds 0.329 for employees with high-level CSR, whereas it becomes negative for those with low-level CSR. Moreover, structural optimization exerts a stronger influence on employees’ social insurance (with a coefficient > 0.792) for employees in high-level CSR corporations, but this influence weakens to a coefficient < –0.054 for low-level CSR employees. For the other two tourism upgrading-social upgrading relationships, their strength also changed dramatically. Specifically, the effect coefficients of service upgrading on employment improvement and social insurance are greater than 0.274 and 0.355, respectively, for low-level CSR employees, whereas they are less than 0.042 and 0.073, respectively, for high-level CSR employees. In summary, these results provide new empirical evidence for understanding the critical role of the governance environment—particularly government responsibility and corporate social responsibility—in shaping social upgrading outcomes.

### 4.4 Effects of socio demographics

The results related to the effects of socio-demographics on social upgrading are presented in Table 5. As expected, age, education, and local residence were found to be significant determinants shaping tourism industry workers’ social upgrading. Specifically, age was positively related to employment improvement, suggesting that older tourism industry employees are more likely to perceive an improvement in employment compared to younger employees. In addition, tourism employees with higher education were found to demonstrate a greater satisfaction with their social insurance; however, they reported being less satisfied in social dialogue. This is understandable, as employees with higher education are more likely to work in large or state-owned enterprises, which typically have a more robust social insurance system and a more bureaucratic management structure compared to small businesses. Furthermore, local residents were positively related to labor rights, indicating that labor rights are better protected for local individuals. However, no significant differences in social upgrading were observed among employees based on gender, monthly income, or employment duration.

**Table 5:** Effects of social demographics on social upgrading.

From	To			
	Employment improvement (EI)	Social insurance (SI)	Labor rights (LR)	Social dialogue (SD)
Age	**0.049** ^**c**^	0.048	−0.021	−0.055
Gender(male = 1)	0.043	−0.023	0.021	−0.063
Education	−0.007	**0.082** ^**b**^	0.009	**−0.104** ^**a**^
Monthly income	0.010	0.012	0.040	0.003
Local people	0.016	0.030	**0.259** ^**a**^	0.108
Employment duration	0.000	0.035	0.012	0.025

Notes: ^a^significantly different from zero at p < 0.01; ^b^significantly different from zero at p < 0.05; ^c^significantly different from zero at p < 0.10 [55]. All effects are unstandardized.

## 5. Conclusion and discussion

An extensive body of literature has investigated whether and how economic upgrading influences social upgrading in various industries in developing countries [7,8,12,58]. However, very few studies have explored the impacts of tourism industry upgrading on social upgrading, and quantitative research on this topic remains limited. Therefore, the present study contributes to the literature by providing an original empirical investigation focused on the tourism sector in Chongqing, China. Furthermore, to the best of our knowledge, this study is the first to apply a structural equation modeling (SEM) approach to examine both the relationships between economic and social upgrading and the moderating role of the governance environment.

The results of the SEM model highlight the significant impacts of tourism industry upgrading on social upgrading. It was found that tourism facility upgrading, tourism service upgrading, and tourism structural optimization tend to significantly improve social upgrading of tourism industry workers. However, tourism product upgrading seems to have no significant impact on social upgrading. Moreover, the SEM model results also underscore the significant moderating effects of the governance environment. An analysis of how different levels (high, neutral, and low) of the moderating variables (GR and CSR) affect the nature and/or strength of the relationship between tourism industry upgrading and social upgrading reveals that the magnitudes of these impacts vary dramatically at low and high levels of GR and CSR. Our results support the general view reported by most previous studies that economic upgrading usually can promote social upgrading to some extent [7,9,10]. Besides, in line with previous studies [12,17,18], our results also confirm that varying governance environments may lead to variations in the economic-social upgrading relationships. In conclusion, our findings not only demonstrate how tourism industry upgrading influences the social upgrading of related workers but also highlight the significant role of the local governance environment in shaping these outcomes.

The findings of this study have significant policy implications. Firstly, given the significant role of tourism industry upgrading in promoting social upgrading, it is suggested that local governments in Chongqing attach greater importance and take measures to further advance tourism industry upgrading initiatives. Measures such as developing tourism master plans, increasing financial investment in tourism industry development, implementing more favourable tourism policies, and enhancing tourism infrastructure development are potential ways to promote tourism upgrading and, in turn, social upgrading. In conclusion, our results may provide another empirical justification for the massive policy measures in advancing tourism development to enhance social upgrading in China and many other developing countries [59–61]. Moreover, given that upgrading tourism facilities, enhancing service quality, and optimizing structures tend to be more effective in driving social upgrading compared to upgrading tourism products, policy measures that devote more efforts and public resources toward facility upgrading, service enhancement, and structural optimization, rather than product upgrading, are likely to yield greater effectiveness in promoting social upgrading. In addition, the direct effects and moderating effects of the governance environment (GR and CSR) in shaping social upgrading results suggest that improving the governance environment (GR and CSR) may effectively promote social upgrading and enhance the promoting effect of tourism industry upgrading on social upgrading. Specifically, it is suggested that policies designed to enhance the governance level of local governments and drive them to become more supportive of tourism industry development and more accountable for labor rights may effectively promote social upgrading. Local governments’ effective guidance and promotion of CSR construction through formulating policies, providing incentives, and establishing regulatory mechanisms may help to promote social upgrading. Furthermore, the government should strengthen the combination of policy tools, especially facility upgrading policies, service upgrading policies, and policies designed to improve the governance environment, so as to effectively enhance the promoting effect of tourism industry upgrading on social upgrading. On the contrary, if the relevant policy tools do not coordinate with each other, the promoting effect of tourism upgrading on social upgrading may be significantly weakened.

The present study can be extended in the future along several directions. Firstly, since the data used here are cross-sectional, they are only capable of revealing associations between variables, not causality. To establish causal links between tourism industry upgrading, governance environment and social upgrading, a longitudinal approach may be an ideal design for future studies [62]. Secondly, the analysis adopted subjective measures of the variables of tourism industry upgrading and social upgrading. Though subjective measures can effectively capture changes in both the explicit and the implicit components of the variable being measured, they do suffer from many systematic biases [63]. Therefore, in future studies, a combination of subjective and objective measurement methods can be adopted to improve the accuracy of the results. Thirdly, since the analysis is based on data from Chongqing, China, the findings can only be applied to the southwest region of China, where the economic and governance environment is similar. To gain a comprehensive understanding of the intricate relationship between tourism industry upgrading and social upgrading, more empirical studies on such relationships in different geographical contexts are needed in the future. Lastly, to gain deeper insights into the mechanisms driving tourism and social upgrading, future studies might integrate qualitative methods—such as in-depth interviews—alongside the quantitative analysis used here.

## Declaration of generative AI and AI-assisted technologies in the writing process

During the preparation of this work the author(s) used WeTab AI Pro in English editing to improve readability. After using this tool/service, the author(s) reviewed and edited the content as needed and take(s) full responsibility for the content of the publication.

## Supporting information

S1 File Chongqing Tourism upgade and soical upgrade. (XLSX)
